# Gut Microbiota: Novel Therapeutic Target of Ginsenosides for the Treatment of Obesity and Its Complications

**DOI:** 10.3389/fphar.2021.731288

**Published:** 2021-08-27

**Authors:** Tongxi Zhuang, Wei Li, Li Yang, Zhengtao Wang, Lili Ding, Mingmei Zhou

**Affiliations:** ^1^ Shanghai Key Laboratory of Complex Prescriptions and MOE Key Laboratory for Standardization of Chinese Medicines, Institute of Chinese Materia Medica, Shanghai University of Traditional Chinese Medicine, Shanghai, China; ^2^ Center for Chinese Medicine Therapy and Systems Biology, Institute for Interdisciplinary Medicine Sciences, Shanghai University of Traditional Chinese Medicine, Shanghai, China; ^3^ Shanghai R&D Center for Standardization of Traditional Chinese Medicines, Shanghai, China

**Keywords:** obesity, gut microbiota, ginsenosides, mechanism, target

## Abstract

Obesity, generally characterized by excessive lipid accumulation, is a metabolic threat worldwide due to its rapid growth in global prevalence. Ginsenosides are crucial components derived from natural plants that can confer metabolic benefits for obese patients. Considering the low bioavailability and degradable properties of ginsenosides *in vivo*, it should be admitted that the mechanism of ginsenosides on anti-obesity contribution is still obscure. Recently, studies have indicated that ginsenoside intervention has beneficial metabolic effects on obesity and its complications because it allows for the correction of gut microbiota dysbiosis and regulates the secretion of related endogenous metabolites. In this review, we summarize the role of gut microbiota in the pathogenetic process of obesity, and explore the mechanism of ginsenosides for ameliorating obesity, which can modulate the composition of gut microbiota by improving the metabolism of intestinal endogenous substances and alleviating the level of inflammation. Ginsenosides are expected to become a promising anti-obesity medical intervention in the foreseeable clinical settings.

## Introduction

Obesity, a complicated chronic metabolic disorder (An et al., 2020), causes visual changes in humans, alongside the excessive accumulation of adipose tissue (Hammarstedt et al., 2018; Ruiz-Ojeda et al., 2019). Long-term imbalance between calorie intake and metabolic consumption is the main cause of obesity, which can also lead to increases in systemic inflammation levels and excessive oxidative stress (Karam et al., 2017; Brooks-Worrell and Palmer, 2019). A strong correlation exists between obesity and complications like diabetes, nonalcoholic fatty liver disease (NAFLD) (Xiao et al., 2017) and increased mortality (Jung and Choi, 2014; Leoni et al., 2018; Tate et al., 2020). The menace of obesity and its complications within our already overburdened healthcare systems is staggering.

**FIGURE 1:**
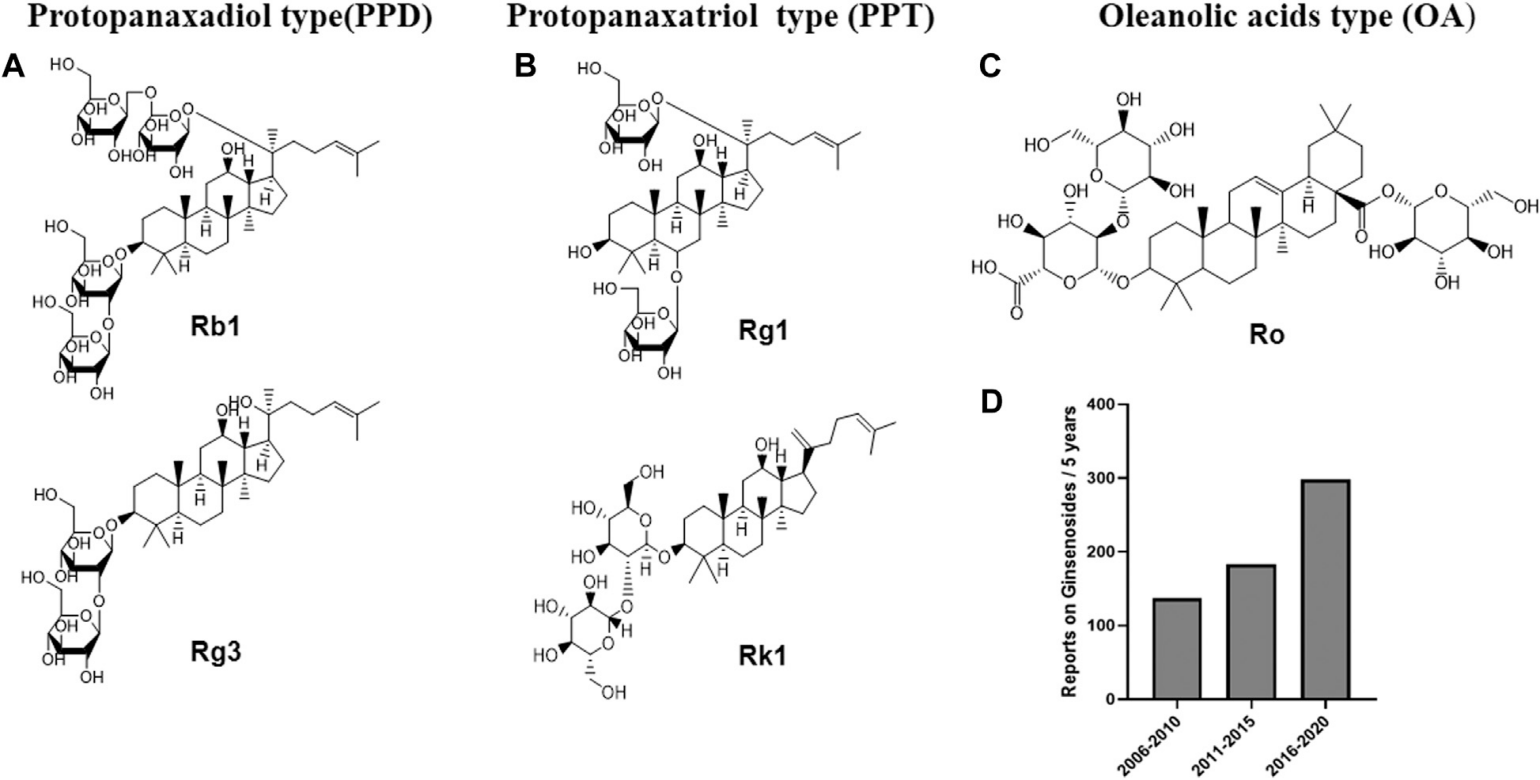
Chemical structures of representative primary ginsenosides from ginseng for improving obesity and its complications targeting at gut microbiota. **(A)**. Protopanaxadiol type(PPD), **(B)**. Protopanaxatriol type (PPT), **(C)**. Oleanolic acids type (OA), **(D)**. Literature deposited in the PubMed database from 2006 to 2020 using ginsenosides, obesity and complications as subject terms.

Ginsenosides, commonly attributed to triterpenoid saponins in botany, mainly exist in the plants of ginseng, *Korean* ginseng and *Panax notoginseng.* (Kim D. et al., 2018; Zhang et al., 2021). Ginsenosides are generally divided into three forms (Figure 1): oleanolic acid pentacyclic triterpenoid saponin Ro, which is exceedingly low in concentration and thus rarely detected; as well as panaxadiol saponins (such as Rb1, Rb2, Rc, Rd, F2, Rg3, Rh2, etc.) and panaxatriol saponins (such as Re, Rg1, Rg2, Rh1, etc.), both of which are dammarane-type tetracyclic triterpenoid saponins (Sun et al., 2006; Choi, 2008). Modern pharmacological studies have suggested that ginsenosides have good anti-tumor, anti-inflammatory, antioxidant and anti-apoptosis properties (Shibata, 2001; Kim et al., 2017; Liu et al., 2019; Yao et al., 2019). In addition, recent studies have shown that multiple ginsenosides improve obesity by regulating different targets. For instance, ginsenoside Mc1 has been shown to ameliorate glucose intolerance and insulin resistance by suppressing c-Jun N-terminal kinase (JNK) phosphorylation (Roh et al., 2020). Ginsenoside Rb1 extracted from crude saponins of Korea red ginseng has also become a promising product for the treatment of obesity and related metabolic disorders by modulating peripheral and central appetite-regulating signals (Park et al., 2019). Ginsenoside Rg3 promoted the browning of white fat in obese mice, as well as significantly ameliorating body weight and blood lipid profiles (Mu et al., 2021). Meanwhile, it is worth noting that, in two clinical investigations, Korean red ginseng failed to improve the insulin sensitivity of non-diabetic healthy overweight and obese adults, and oral ginsenoside Re therapy was unable to upregulate insulin sensitivity in overweight and obese subjects with impaired glucose tolerance or newly diagnosed diabetes, which indicated that certain ginsenosides or ginseng processing drug may have no significant effect on the recovery of insulin sensitivity in obese patients (Reeds et al., 2011; Cho et al., 2013). In summary, although the efficacy of Ginsenoside in improving insulin sensitivity is questionable, ginsenosides show strong potential for regulating glucose and lipid metabolism, and thus conferring metabolic benefits on obesity.

With increased understanding of gut microbiota and metabolomics in recent years, we now know that onset of metabolic diseases is closely related to changes in gut microbiota composition (Devaraj et al., 2013; Mei et al., 2016; Kootte et al., 2017). Gut microbiota is commonly considered to be indispensable normal microorganisms that reside in the intestine. Different forms of bacterial subtypes restrict and depend on one another in order to form a qualitative and quantitative ecological balance. Thus, gut microbiota constitutes a balanced system, and participate in many physiological and pathological processes (Ley et al., 2006; Saad et al., 2016; Pickard et al., 2017). In the past few decades, studies have shown that gut microbiota dysfunction might induce obesity and provoke serious stress responses (Tsai and Coyle, 2009). Ley first identified the correlation between obesity and gut microbiota, finding that *Bacteroides* decreased in obese mice, but that increases in *Firmicutes* occurred in the same model (Ley et al., 2005). Subsequent research on *Enterobacter cloacae* in the gut microbiota showed that its overgrowth in the human intestinal tract directly led to obesity (Keskitalo et al., 2018), suggesting a causal link between gut microbiota and obesity. Thus, gut microbiota may be a noteworthy target for the treatment of obesity.

**FIGURE 2:**
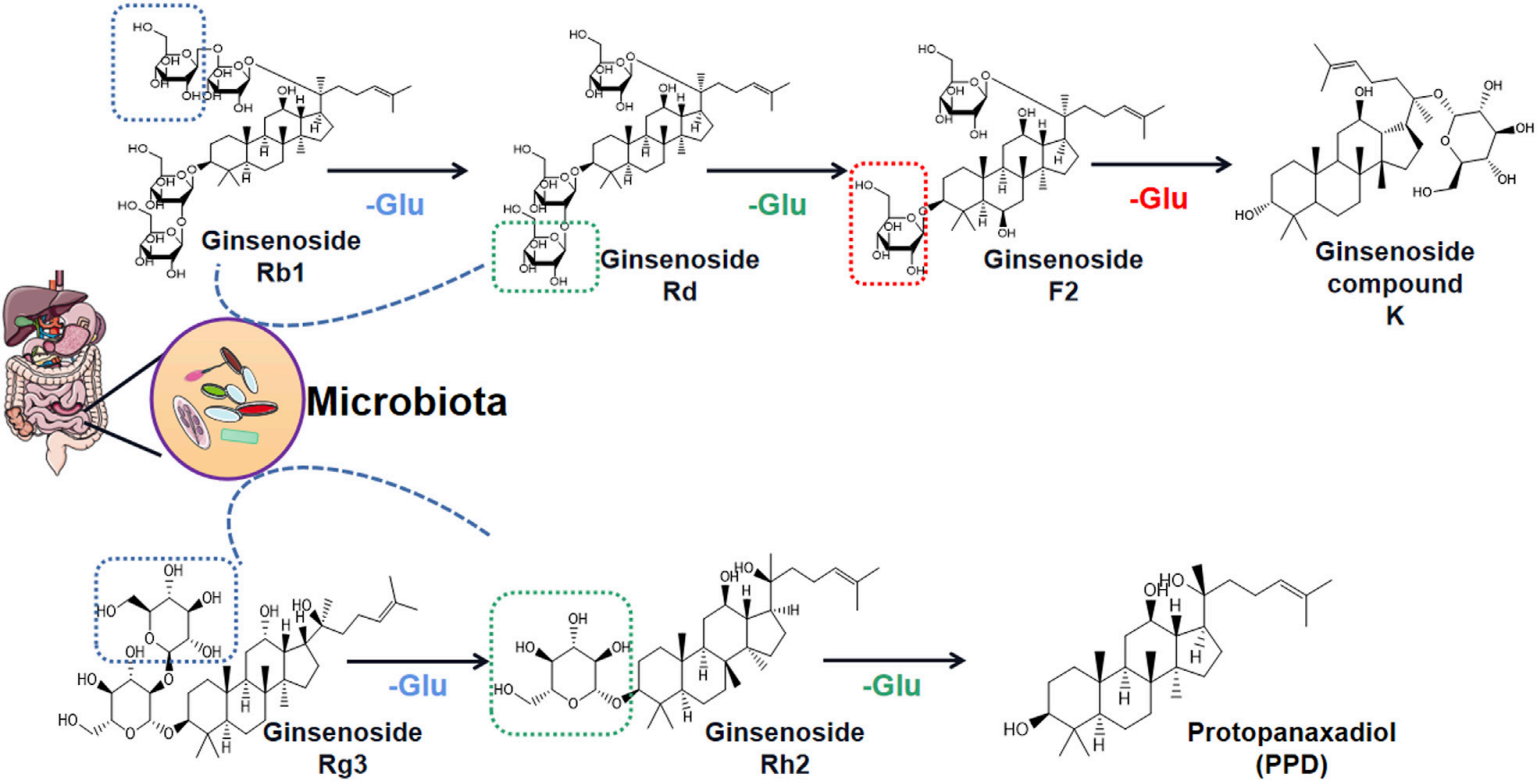
Deglycosylation process of ginsenosides Rb1 and Rg3 through gut microbiota regulation.

An interesting phenomenon has been discovered is that the obesity-improving effects of ginsenosides, which work by acting on the gut microbiota, may be bidirectional. On one hand, some reports have shown that ginsenosides regulate gut microbiota disorders by restoring normal structural composition and thereby improving the obesity phenotype. Gut microbiota is a novel therapeutic target of ginsenosides, and it is expected that ginsenosides will be promoted as a clinical drug for obesity and its complications. On the other hand, the content of ginsenosides in ginseng species is extremely rare, and most ginsenoside monomers have inherent clinical disadvantages, such as low water solubility, fast elimination rate, and low bioavailability (Pan et al., 2018; Yang et al., 2020). So how do they exert their weight loss effects? In recent years, many studies have revealed that ginsenosides are biodegraded by gut microbiota and are metabolized into monoglycosides and aglycons through deglycosylation reactions (Figure 2) (Akao et al., 1998; Li R. et al., 2018). Thus, both ginsenosides and gut microbiota may have complementary, symbiotic effects on obesity rather than their interaction being a case of simple target action.

In this review, we clarify the mechanisms of gut microbiota for addressing the pathophysiology of obesity. In addition, we describe the significance of gut microbiota as a target for ginsenosides’ anti-obesity effects, in order to enrich future theoretical research on the mechanism of ginsenosides in the context of their clinical application, and also to provide new insight around the power of natural products for ameliorating metabolic diseases.

## The Role of Gut Microbiota Dysbiosis in the Pathophysiological Mechanism of Obesity

**FIGURE 3:**
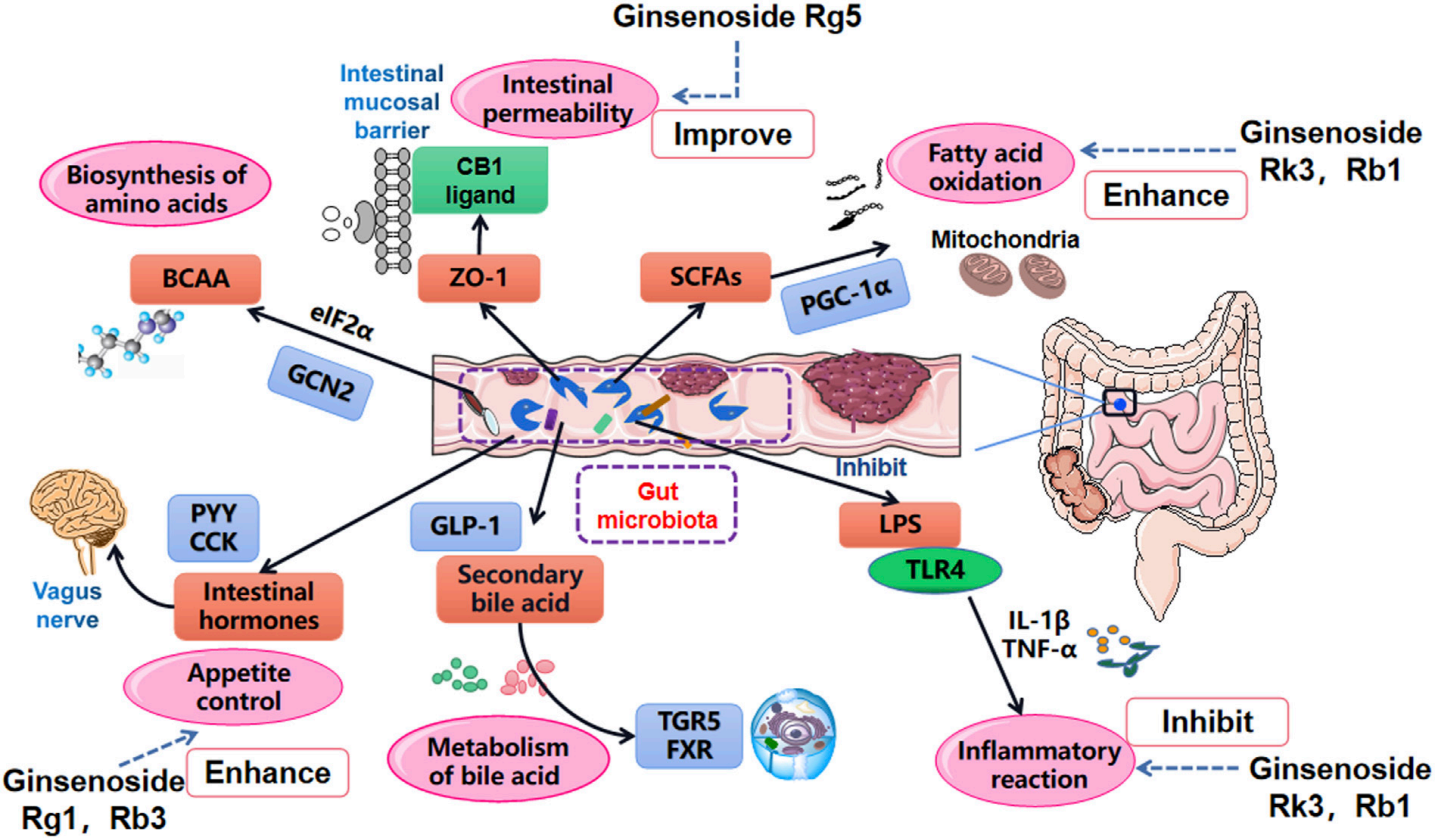
Main influential pathways for obesity improvement *via* the role of gut microbiota manipulation. Gut microbiota affects the oxidation of SCFAs, the absorption of LPS and inflammation level, changes intestinal mucosal permeability by acting on tight junction protein ZO-1, manages the metabolism of bile acids by regulating TGR5 and FXR, influences the metabolism of BCAA and release of intestinal hormones.

Diet is an indispensable external factor that affects gut microbiota composition (David et al., 2014; Sonnenburg and Bäckhed, 2016). The primary initiators of obesity are decreased exercise and increased intake of high-energy food. In addition, various gene polymorphisms have significant effects on the pathogenesis of obesity (Huang et al., 2018; Kim HJ. et al., 2018). Specific proteins and hormone factors play a role in regulating metabolism and body weight. The composition of human intestinal microflora is transformed when diets are changed (Scott et al., 2013; Gentile and Weir, 2018). Consequently, the alteration of gut microbiota will impact fatty acid metabolism, affecting multiple inflammatory factors signaling pathways and even the original environment of the intestinal tract. After this, deposition of massive adipose tissue will emerge, eventually damaging the host’s homeostasis (Figure 3) (Kobyliak et al., 2016; Hersoug et al., 2018; Torres et al., 2019).

### Gut Microbiota Dysbiosis Can Inhibit Short Chain Fatty Acids Oxidation

Short chain fatty acids (SCFAs) are some of elementary substances involved in the synthesis of human adipose tissue. They also serve an important role in the obesity process by regulating triglycerides, adipocyte differentiation, and leptin expression (Tan et al., 2014; De la Cuesta-Zuluaga et al., 2019; Jiao et al., 2020). Once gut microbiota falls into a state of disorder, SCFAs are spontaneously influenced by varying proportions of *Bacteroides* and *Pachytene*, resulting in decreases in butyrate and crippled expression abilities of G protein-coupled receptor 41 (GPR41) and G protein-coupled receptor 43 (GPR43) (Ang and Ding, 2016; Lu et al., 2016). The oxidation process of SCFAs can be reversed, however, by inhibiting the phosphorylation of peroxisome proliferator-activated receptor *γ* coactivator 1α (PGC-1α), and inhibiting the expression of mitochondrial uncoupling protein-1 (UCP-1) in brown adipose tissue (BAT) (Argentato et al., 2018; Wang et al., 2020). In addition, research has shown that gut flora partly inhibits intestinal fasting-induced adipocyte factor (FIAF) by promoting agonistic activity of the lipoprotein lipase in adipocytes and thus decreasing lipid deposition and storage (Bäckhed et al., 2004; Aronsson et al., 2010; Guo et al., 2017). Increased FIAF has also been shown to stimulate the production of PGC-1α, upregulate the expression of the fatty acid oxidation gene in mitochondria, and complete fatty acid oxidation (Alex et al., 2013; Xiao et al., 2019).

### Gut Microbiota Dysbiosis Can Increase the Absorption of Lipopolysaccharide and Cause Inflammation

LPS (lipopolysaccharide) is a common component of Gram-negative bacteria in the gut microbiota. Researchers have identified that LPS levels in rats fed with a high-fat diet were significantly higher than those in the control group, and that rats in the high-fat diet group existed in a sustained low-grade inflammation state (Bernard et al., 2019; Zhang H. et al., 2020). Cani named the host inflammatory response induced by low level LPS as “metabolic endotoxemia” (Cani et al., 2007). He believed that LPS was one of the major risk factors for metabolic disorders, and helped explain why there was a causal link between gut microbiota and obesity (Cani et al., 2008; Everard et al., 2013). Further study found that this LPS-mediated inflammation could be reversed. The obesity metabolic phenotype of mice was significantly improved when CD14 was eliminated, and when antibiotic interventions were used to reduce the inflammatory state caused by lipopolysaccharides (Carvalho et al., 2012; Boutens et al., 2018).

Increased LPS levels may be related to obesity. Certain diets can induce leptin resistance in the vagus nerve afferent of obese mice, and LPS is believed to play a role in this resistance (De Lartigue et al., 2011). Gut microbiota is involved in the inflammatory and obesity process by stimulating host appetite (Torres-Fuentes et al., 2017). LPS-induced obesity has also been causally linked to the binding of scavenger receptor type B (SR-BI) (Hersoug et al., 2016). These binding products enhanced LPS’ actions (i.e., promoted the transport of lipoproteins on the endothelial barrier and the endocytosis of adipocytes), accelerated the transformation of macrophages from M2 type to M1 type in adipose tissue, and mediated inflammatory reactions (Xiong et al., 2018; O’Reilly et al., 2019). In addition, it has been shown that LPS contributes to the expression of receptor protein mRNA that is related to angiotensin receptors, and that this expression was positively correlated with specific bacteria in the gut (Oliveira et al., 2020).

### Gut Microbiota Dysbiosis Can Change Intestinal Mucosal Permeability

Intestinal homeostasis is a dynamic balance formed by the interactions between the intestinal mucosal barrier and the gut microenvironment and its metabolites. The intestinal mucosal barrier prevents the invasion of pathogenic antigens and maintains intestinal health (Martens et al., 2018). Gut microbiota plays a crucial role in influencing tight junctions (TJ) and intestinal epithelial permeability to enhance the stability of intestinal epithelial cells.

Cumulative high-fat diets induce lesions in the composition of gut microbiota, and thus are also linked to intestinal epithelial damage. Gut microbiota disorders can activate intestinal epidermal growth factors, which directly act on protease activated receptors, cripple intestinal TJ structure, significantly down-regulate the expression of TJ protein zonula occludens 1 (ZO-1), eventually destroying the structure of intestinal barrier and increasing the amount of lipopolysaccharide entering the blood from the intestinal tract (Guo et al., 2013; Pontarollo et al., 2020). It has been found that gut microbiota affects the permeability of intestinal barrier partly *via* the actions of glucagon like peptide 2 (GLP-2) (Cani et al., 2009). When the proportion of *Lactobacillus* and *Bifidobacterium* in the intestinal tract is decreased, the production of intestinal GLP-2 is also reduced. GLP-2 has also been shown to weaken the tight connection between intestinal epithelial cells, increase the amount of lipopolysaccharides in the blood, and ultimately aggravate the inflammatory system (Watson and Duckworth, 2010). By employing prebiotics to maintain the quantity of *Bifidobacteria* in the host gut, gut barrier damages can be reversed: mice treated with prebiotics showed resistance to endotoxin level increases and obesity that would have been induced by high-fat diets (Krumbeck et al., 2018). Kuhn found that intraepithelial lymphocytes (IEL), a main IL-6 producer, are located at the epithelial barrier and maintain intestinal epithelial homeostasis (Regner et al., 2018). Bacteria in *Bacteroides* promote the presence of IEL in the colon and reduce the integrity of the intestinal epithelium by reducing the expression of blocked protein 1 and thinning the gel layer (Kuhn et al., 2018).

### Gut Microbiota Dysbiosis Can Change the Metabolism of Bile Acids

Bile acids (BAs) are a crucial component of bile, and regulate lipid metabolism by controlling the activity of pancreatic lipase and lipoprotein esterase, and by improving the metabolism of fat hydrolysis. BAs are also responsible for lipid transport in the gut and fat absorption (Chávez-Talavera et al., 2017). On the whole, routine BA manipulation is inseparable from regulation of the gut microbiota. Former research has shown that mice treated with antibiotics showed increased intestinal bile acid reabsorption, and significantly lowered bile acid excretion in their feces (Behr et al., 2019). Farnesoid X receptor (FXR) and G protein coupled bile receptor 5 (TGR5) are classic bile acid receptors transformed by intestinal microorganisms, which are responsible for the balance of bile acid, lipid and glucose homeostasis in the host liver, intestine and peripheral tissues (Chiang, 2017; Jadhav et al., 2018). Researchers have found that Berberines changed gut microbiota physiology, as well as the composition and function of the bacteria. In particular, it reduced the activity of the bile salt hydrolase expressing bacteria *Clostridiumspp*, which altered bile acid metabolism and activated FXR signaling (Tian Y. et al., 2019). Another study found that acetylation factors and Bacteroides were increased in the intestine of mice treated with fexaramine (an FXR agonist). This process increased the secretion of taurolithocholic acid (TLCA), fibroblast growth factor 15 (FGF15) and glucagon like peptide 1 (GLP-1), which stimulated TGR5 expression in the intestine, promoted the secretion of GLP-1, improved insulin and glucose tolerance, and promoted the browning of white adipose tissue in mice (Pathak et al., 2018).

### Gut Microbiota Dysbiosis Can Suppress the Metabolism of Branched Chain Amino Acids

Branched chain amino acids (BCAAs, i.e., leucine, isoleucine, and valine) are essential amino acids. They are commonly obtained from the diet, are elementary components for muscle tissue construction, and are essential amino acids for normal human activity (Wu, 2013). BCAAs are not only closely related to fatty acid synthesis, glucose transport and intestinal function regulation, but are also involved in the regulation of energy consumption and the prevention of early chronic metabolic diseases (Yoneshiro et al., 2019). Compared with the original gut microbiota composition, BCAA levels in patients with gut microbiota metabolic disorders is generally downregulated (Zeng et al., 2020). In other words, disorders of bacterial community structure inhibit the synthesis and/or release of BCAAs. Disordered gut microbiota initiated decreases in general controlled nonderepressible 2 (GCN2) kinase levels, hindered the phosphorylation of eukaryotic translation initiation factor 2α (eIF2α), and inhibited the biosynthesis of amino acids, thus contributing to the occurrence of endoplasmic reticulum stress. Eventually, chronic inflammation and obesity take place in the human body (Tian M. et al., 2019; Golonka et al., 2020). Additionally, other studies have shown that BACC also improved the abundance of gut microbiota. Leucine and isoleucine regulate the expression of lipid metabolism genes *via* SIRT1 and the lipolytic gene AMP-activated protein kinase (AMPK), increase the abundance of *Lachnospiraceae*, decrease the abundance of *Desulfovibrionaceae* and relieve lipid deposition (Ma et al., 2020). Asis reported that BCAA and gut microbiota mutually regulate obesity (Saad et al., 2016; Yue et al., 2019; Zhang L. et al., 2020): Gut microbiota affect the synthesis and metabolism of BCAA by acting on signaling pathways, but the reduction of BCAA biosynthesis will lead to chronic inflammation in the body, which can aggregate obesity indicators and cause a serious imbalance in gut microbiota proportions.

### Gut Microbiota Dysbiosis Can Affect the Release of Gut Hormones

Gut hormones are peptides that are released into circulation by endocrine cells and gastrointestinal tract neurons. They not only regulate the secretion of digestive glands and alimentary canal movements, but are also responsible for the release of other hormones (Gribble and Reimann, 2019). As mentioned earlier, the final product of indigestible dietary fiber which is fermented by colon gut microbiota is SCFAs. The release of diverse intestinal hormones is closely related to SCFA metabolism. For example, acetate enters the brain through the blood-brain barrier and blood circulation, activates the parasympathetic nervous system, promotes islet B cells to secrete large amounts of insulin, and induced glucose conversion into ATP. In addition, it promoted the release of ghrelin from the stomach, which triggered eating in order to alleviate hunger sensations. Over time, individuals with imbalanced gut microbiota continued to increase their food intake, thus increasing the risk of both insulin resistance and obesity. Peptide YY (PYY) is a kind of intestinal hormone expressed in digestive tract L cells which are released into blood circulation following eating. PYY significantly reduced food intake, which also relieved adipose deposition and improved obesity symptoms (Lafferty et al., 2018). SCFAs increases caused by gut microbiota imbalances have been shown to combine with the actions of the GPR41 receptor to promote the secretion of PYY by terminal ileum L cells (Duca et al., 2013). PYY slowed down intestinal peristalsis, prolonged the time of food passing through the intestine, promoted the absorption of nutrients, and increased lipid accumulation. Therefore—as the gut microbiota affected the synthesis and release of SCFAs, its regulatory effects on intestinal hormones [(i.e., GLP-1 and cholecystokinin (CCK)], were paralleled by those of PYY (Diamant et al., 2011; Perry and Wang, 2012). The gut microbiota targeted these intestinal hormones, which therefore affected obesity occurrence. This is not only a novel factor influencing the link between obesity and abnormal gut microbiota composition, but also represents a potential new breakthrough for future obesity treatment.

## Ginsenosides Act as Anti-Obesity Agents by Modulating the Gut Microbiota as a Novel Target

The anti-obesity effects of ginsenosides are tightly connected with their regulatory role on gut microbiota. When ginsenosides are metabolized in the intestine, they significantly control the circulating levels of gut hormone metabolites that are related to obesity, improve the intestinal microenvironment, and enhance the barrier function of the intestinal tract. In recent years, several reports have suggested that ginsenosides family could improve outcomes related to obesity and its comorbidities by modulating flora functions and restoring the structure of gut microbiota (Table 1) (Li J. et al., 2018; Wei et al., 2020). This is undoubtedly a promising target for the treatment of metabolic disorders caused by obesity.

**TABLE 1:** Ginsenosides significantly improve obesity and its complications by regulating gut microbiota.

Diseases	Compounds/extracts	Subject	Gender	Period	Main gut microbiota analysis	Mechanism	References
Obesity	Ginsenoside Rb1	Mice	Both Genders	4 weeks	The abundance of *Clostridia* and *Lactococcus lactis* increased	Enhance the production of acetate and butyrate contents of all SCFA	Hasebe et al. (2016)
Obesity	Ginsenoside Rb1	Mice	Male	8 weeks	The relative abundance of *Firmicutes* phylum increased and the relative abundance of *Bacteroidetes* phylum improved	Reduce the overall diversity of the gut microbiota in feces and change the microbial composition	Bai et al. (2021)
Obesity	*Panax notoginseng* saponins	Mice	Male	7 Weeks	The abundance of *Akkermansia muciniphila* and *Parabacteroides distasonis* increased	Activate leptin AMPK/STAT3 signaling pathway to promote BAT thermogenesis and beige adipocyte reconstruction	Xu et al. (2020b)
Obesity induced colitis	Ginsenoside Rk3	Mice	Male	8 weeks	The relative abundance of *Actinomycetes* and *Clostridiathat*, *Parabacteroides*, *Lactobacillus*, *Butyricicoccus* and *Clostridium* increased, while that of *Akkermansia*, *Acetobacte*r, *Enterobacter*, and *Anaerotruncus* decreased	Inhibit TLR4/NF-κB signaling pathway, and improve the metabolic imbalance of intestinal flora, as well as significantly reduce the ratio of *Firmicum*/*Bacteroide* and relieve the inflammatory cascade	Chen et al. (2021)
Diabetes	Ginsenoside T19	Mice	Male	6 weeks	The value of *Firmicutes*/*Bacteroidetes* decreased and the relative abundance of the *Lachnospiraceae* family remarkably raised	Lower the levels of blood glucose and lipid, alleviate insulin resistance *via* AMPK and PI3K Pathways	Xu et al. (2020a)
Diabetes	Ginsenoside Rg5	Mice	Male	4 weeks	The abundance of *Firmicutes* and *Verrucomicrobia* decreased at the phylum level, and the abundance of *Bacteroidetes* and *Proteobacteria* increased	Repair intestinal barrier function and relieve metabolic endotoxemia-related inflammatory pathways	Wei et al. (2020)
Diabetes	Ginsenoside Rb1+ Ginseng polysaccharides (GP)	Rats	Male	30 days	The abundance of *β*-D-glucosidase producing probiotics *Bifidobacteria* spp. *Bacteroides* spp. and *Lactobacillus* spp. showed no significant change	Regulate intestinal flora, improve fecal *β* - D-glucosidase activity, and improve the conversion rate of ginsenoside Rb1 to CK	Li et al. (2018a)
NAFLD	Ginsenoside Rg1 + Rb1 + Rg3	Human	Both Genders	4 weeks	The abundance of *Lactobacillus* significantly increased	Improve liver enzymes (alanine aminotransferase) and fatigue score by modulating gut microbiota	Hong et al. (2020)
NAFLD	Ginsenoside extract	Mice	Male	12 weeks	The abundance of *Bacteroidetes* significantly increased and the ratio of *Firmicutes* to *Bacteroidetes* down-regulated	Enhance the gut barrier function, restore the energy balance, and alleviate metabolic inflammation by gut microbiota regulation	Liang et al. (2021)

Note: Human studies in the table were randomized controlled clinical trials.

### Ginsenosides Have Intervention Effects on Obesity by Promoting Short Chain Fatty Acids Metabolism

SCFAs, known as the “secret weapon” of gut microbiota, participate in the process of material absorption and lipid metabolism (Den Besten et al., 2013, 2015; Canfora et al., 2015). The dietary fiber consumed by organism is not digested directly by intestinal tract, but by SCFAs to complete digestion engineering; Meanwhile, the production of SCFAs may increase with this dietary fiber intake. SCFAs play a large role in maintaining the normal functions of the large intestine, as well as the morphology and function of colon epithelial cells. They also promote the synthesis and accumulation of lipids in hosts, which can lead to obesity once fatty acid metabolism is blocked (Kasubuchi et al., 2015; Figueiredo et al., 2017; McNabney and Henagan, 2017). Ginsenoside Rk3 administration has been demonstrated to significantly attenuate weight loss, increase SCFAs levels (including acetic acid, butyric acid and isovaleric acid), protect intestinal barrier function, and blockade the Nucleotide-binding oligomerization domain-like receptor 3 (NLRP3) inflammasome pathway (Tian et al., 2020). Fermented ginseng has also been shown to promote SCFA oxidation and decomposition, which significantly improves the capability of SCFAs to produce *Akkermansia* (which is in the *Verrucomicrobia* phylum) (Fan et al., 2019). Another study found that the conversion of ginsenoside Rb1 to ginsenoside compound C increased the number of bacterial genera (including *Clostridia* and *Lactococcus lactis*), and enhanced the acetate and butyrate contents of all SCFAs in the cecum and feces (Hasebe et al., 2016). It is worth noting that, although most studies focus on the short-chain fatty acids produced by the microbiota, long-chain fatty acids, often in the form of esters, are also important gut microbiota metabolites. It has been found that *Enterotoxigenic Escherichia coli* can block the uptake of long-chain fatty acids (LCFAs) into intestinal epithelial cells by activating the phosphorylation of peroxisome proliferator activated receptor *γ* (PPAR-γ) (Li et al., 2019). Ginsenoside extract (GE) has also been shown to be capable of inducing *Enterococcus faecalis* to produce unsaturated LCFA and mystic acid. This action significantly improved obesity by activating brown adipose tissue (BAT) and promoting formation of beige fat (Quan et al., 2020).

### Intervention Effects of Ginsenosides on Obesity by Improving Inflammation Reactions

Chronic inflammation caused by lipid accumulation is mainly mediated by LPS stimulating immune reactions and the release of pro-inflammatory factors. LPS, a component of Gram-negative bacteria in the gut, is constantly produced in the intestinal tract through the death of Gram-negative bacteria (Rietschel et al., 1994). LPS enters the plasma and is transported by lipopolysaccharide binding protein (Hersoug et al., 2018; Molinaro et al., 2020). The most general change related to LPS is the increased expression of inflammatory factors, such as IL-1, IL-6, TNF-α, monocyte chemoattractant protein-1(MCP-1) (Seifert et al., 2015). These pro-inflammatory factors cause the structural destruction of gut microbiota, which can then induce systemic inflammation in obese individuals. Ginsenosides dramatically ameliorate obesity induced by high-fat diets *via* regulation of the homeostasis of gut microbiota. Research has suggested that water-extracted white ginseng (WEWG) is rich in diverse high-polar-to-low-polar ginsenosides, which are potentially more suitable for correcting obesity-related gut microbiota disorders. Specifically, WEWG reduced the ratio of *Firmicutes* to *Bacteroidetes* by 65%, and significantly increased the abundance of *Lactobacillus* and *Parabacteroides*. This improved gut microbiota balance could better alleviate systemic inflammation and enteric metabolic disorders—and thus ultimately had anti-obesity effects (Zhou et al., 2020). The ginsenoside Rb1 has been shown to significantly decrease body weight, alleviate fasting blood glucose, improve blood lipid profiles, and decrease inflammation levels in patients when downward trends of gut microbiota diversity in fecal samples. After Rb1 consumption, *Firmicutes* phylum increased and the relative abundance of *Bacteroidetes* phylum improved. At the family level, *Helicobacteraceae* and *Ruminococcaceae* decreased and *Rikenellaceae* were enriched (Bai et al., 2021). In addition, in the model of obesity-induced colitis, ginsenoside Rk3 effectively ameliorated the metabolic dysbiosis of intestinal flora with significantly decreased *Firmicute*/*Bacteroidete* ratios and suppressed the inflammatory cascade by inhibiting the TLR4/NF-κB (Toll-like receptor 4/nuclear factor kappa-B) signaling pathway (Chen et al., 2021).

### Intervention Effects of Ginsenosides on Obesity *via* the Regulation of Gut Hormones

In recent years, research on metabolic diseases, including obesity and diabetes, has focused on understanding gut hormones. Common intestinal hormones include ghrelin and gastrin, which are secreted from the stomach, insulin and glucagon, which are secreted from the pancreas, CCK, ghrelin, gastric inhibitory peptide (GIP) and motilin, which are secreted from the small intestine, as well as PYY and GLP-1, which are secreted from the large intestine. The secretion of these hormones is closely related to changes in the composition of gut microbiota. The ginsenoside Rg3 has been shown to stimulate GLP-1 secretion by activating sweet receptor signals, and the Ginsenoside Rg1 can significantly increase phosphorylation levels of AMPK in epididymal white adipose tissue and 3T3-L1 cells, which thereby inhibits adipogenesis and counters adipose accumulation (Kim et al., 2015; Liu et al., 2018). *Panax notoginseng* saponins (PNS), which includes multiple ginsenosides, has been demonstrated to reshape the murine gut microbiota by increasing the abundance of *Akkermansia muciniphila* and *Parabacteroides distasonis*. This stimulates the reconstruction of beige adipocytes by activating the leptin-AMPK/STAT3 signaling pathway, which ultimately promotes energy expenditure. However, PNS-induced modulation of gut microbiota has negative effects in leptin gene-deficient mice, suggesting that PNS’ obesity improving effects are inseparable from the interaction between intestinal flora and insulin (Xu Y. et al., 2020). Ginsenoside Rg3 is able to stimulate GLP-1 secretion by activating sweet receptor signals, and both the secretion of GLP-1 and the occurrence of inflammation may be related to the composition of gut microbiota. GLP-1 exerts anti-obesity effects by delaying gastric emptying, reducing food intake, stimulating insulin release, improving insulin sensitivity (Van Can et al., 2014), and sending appetite suppressor signals through the sympathetic nervous system. Rg1 has also been shown to significantly increase AMPK phosphorylation levels in epididymal white adipose tissue and 3T3-L1 cells. This inhibits adipogenesis, reduces intracellular lipid content, decreases adipocyte size, and counters adipose accumulation (Kim et al., 2015; Liu et al., 2018). However, there are relatively few reports on gut microbiota as an obesity-improving therapeutic target for ginsenosides, mainly because it difficult to detect specific changes in gut microbiota composition after administration. In addition, the results of different analysis methods adopted in the original data, such as 16S rRNA sequencing and metagenomics, may be quite diverse, which obscures the stability of the results. Therefore, further exploration of these complex mechanism is needed to fully understand how ginsenosides improve obesity symptoms by restoring the dysbiosis of microorganisms.

## Intervention Effects of Ginsenosides on Obesity-Related Complications

There has been a troubling increase in the worldwide prevalence of diabetes as people’s lifestyles (i.e., excessive energy intake and decreased exercise) have become altered (Balakumar et al., 2016). Diabetes is now one of the most common endocrine and metabolic diseases, which can be primarily attributed to evolving obesity and insulin resistance. Thus, diabetes is one of the most important complications of obesity. Insulin resistance and insulin secretion imbalance are the two key pathological characteristics of diabetic patients. Insulin resistance is induced by a long-term imbalance of glucose absorption and metabolism, and interacts with disordered gut microbiota compositions (Yang et al., 2018). Ginsenoside family compounds are a potentially effective drug for treating type 2 diabetes by improving gut microbiota. Ginsenoside T19 is able to effectively lower blood glucose and lipid levels, ameliorate insulin resistance, improve liver and pancreatic histopathology by decreasing *Firmicutes*/*Bacteroidetes* values, and increasing the relative abundance of beneficial *Lachnospiraceae* bacteria regulate carbohydrate metabolism (Xu J. et al., 2020). Studies have shown that the ginsenoside Rg5 significantly relieved inflammation related to metabolic endotoxemia, and repaired the original function of the intestinal barrier. It also appears to significantly reverse gut microbiota malnutrition while reducing the *Firmicutes/Bacteroidetes* ratio significantly. Additionally, Rg5 dramatically reduced the abundance of *Firmicutes* and *Verrucomicrobia* in diabetic mice at the phylum level, and increased the abundance of *Bacteroidetes* and *Proteobacteria*, suggesting that Rg5 can reverse gut microbiota disorders and metabolic disorders related to Type 2 diabetes (Wei et al., 2020). An investigation by Li found that the ginsenoside Rb1 had hypoglycemic activity in diabetic rat model. It worked by promoting *β*-Glucosidase activity, which is mainly produced by *Bififidobacteria*. Ginseng polysaccharides enhanced the hypoglycemic activity of ginsenoside Rb1 and altered its biotransformation pathway, which promoted the transformation of ginsenoside Rb1 to CK. The improved Rb1 bioavailability contributed to hypoglycemic effects by modulating gut microbiota (Li J. et al., 2018). Another study showed that the main reason that Rb1 exposure increases in diabetic rats led to increased Rb1 absorption through the intestinal tract, and helped gut microbiota inhibit the de-glycosylation process, which positively affected the clinical treatment of diabetes (Liu et al., 2015). Generally, gut microbiota promotes the metabolism transformation of ginsenosides, improves their bioavailability, and stimulates the local production of secondary metabolites. Thus, they have a large role in lowering blood glucose and treating diabetes.

In addition to diabetes, ginsenosides have also been applied to treat NAFLD (Nonalcoholic fatty liver disease). In the west, 20–30% of the adult population have NAFLD (Loomba and Sanyal, 2013). One of the major causes of NAFLD is the obesity-induced accumulation of lipids in the liver, which can result in dyslipidemia and symptoms of insulin resistance. Several recent studies have found that the “gut-liver axis” has an influential role in ameliorating NAFLD (Porras et al., 2017; Carbajo-Pescador et al., 2019; Albillos et al., 2020). PNS appears to exhibit potent anti-fibrotic effects in NAFLD mice, and PNS intervention decelerates gut-to-liver translocation of microbiota-derived SCFAs products, which significantly improves the permeability of intestinal mucosa by modulating gut microbiota (Xu et al., 2021). A clinical report revealed that, following administration of Korea Red Ginseng (2,000 mg/day, ginsenoside Rg1 + Rb1 + Rg3 4.5 mg/g) to 45 NAFLD patients over four weeks (Hong et al., 2020), liver alanine aminotransferase levels were significantly improved, and there was a remarkable increase in the abundance of *Lactobacillus*. In addition, *Akkermansia* increased in the group treated with ginsenosides, while other liver markers, including AST, triglyceride, and γ-GT, showed significant decrease when compared with the placebo group. Another research report showed that GE improved the intestine’s mucosal barrier, and exerted anti-inflammatory effects by restoring the composition of gut microbiota in a NAFLD mice model in a dose-dependent manner. Through network analysis, it was shown that GE inhibited NF-κB/IκB signaling and suppressed the release of pro-inflammatory factors. In addition, GE’s improvement effects on NAFLD were inseparable from the promotion of liver lipolysis gene (CPT-1α) expression and amelioration of leptin resistance (Liang et al., 2021).

## Discussion

There are considerable reports that explore the underlying mechanisms of ginsenosides’ anti-obesity effects. However, most of research are concentrated on describing inflammatory reactions or molecular signaling pathways, aiming to illustrate the receptors that ginsenosides activate and potential clinical targets. As an indispensable community of intestinal host, gut microbiota is highly involved in food absorption and metabolism. Because the mechanism of action of ginsenosides for obesity treatment is relatively complicated, we sum up two possible pathways that are mediated by gut microbiota. One possibility is that, in term of the low bioavailability of ginsenosides in the body, it might undergo multiple gut microbiota-mediated deglycosylation reactions in order to produce finalized ginsenosides compounds. These metabolites have beneficial effects on the treatment of obesity and its complications. Another possibility is the gut microbiome itself is a target that cannot be underestimated in the process of improving obesity. Ginsenosides are probably able to regulate the bacteria and rectify the structure of the flora, ameliorating obesity-related complications by promoting the release of related endogenous substances and regulating glucose and lipid metabolism. In summary, the mechanism by which ginsenosides regulate microbiota and improve obesity is still fairly understudied. We are not explicit in whether the effects of ginsenosides on the structure of intestinal flora are direct or indirect, or how secondary metabolites of ginsenosides may play a role in improving obesity. In view of the insufficient research on ginsenosides to alter the abundance of specific bacteria in the process of animal experiment research is not comphesive enough, and few reports on the regulation of gut microbiota by ginsenosides in clinical cases, which restricts clinical application of ginsenosides in obesity, more research should be carried out on these aspects, so as to provide new ideas for the research on the mechanism of ginsenosides improving obesity and its complications.

## Concluding Remarks

Obesity, a chronic metabolic epidemic, has gradually become an issue throughout the world. Accumulating evidence has shown that ginsenosides have anti-obesity effects. Ginsenosides not only improve glucose metabolism, increase insulin sensitivity, and promote lipid transport, but also alleviate symptoms of obesity-related complications like diabetes and NAFLD. Gut microbiota is involved in the pathogenesis of obesity in the fountainhead step, which is responsible for the regulation of fatty acid oxidation, fatty acid synthesis, bile acid release, metabolism of branched chain amino acids, transformation of intestinal permeability, and mediation of a variety of inflammatory reactions. The interaction between ginsenosides and gut microbiota may have far-reaching impacts that improve obesity.

Although targeting microflora with ginsenosides is a technique currently in its infancy, it has the potential to become a clinical choice for the treatment of obesity and other metabolic diseases if thoroughly researched. In the present review, we described several potential pathological mechanisms that could affect gut microbiota and lead to obesity. Next, wanting to further verify that ginsenosides had competent anti-obesity effects, we explored the rationality and necessity of gut microbiota as a novel treatment target. To this end, further research evaluating the feasibility of gut microbiota as a novel target of ginsenosides that has implications for curing obesity and related complications is needed.
